# Editorial: Complex interplay between lung diseases and multisystem disorders: pathogenesis, management, and outcome

**DOI:** 10.3389/fmed.2026.1963551

**Published:** 2026-09-08

**Authors:** Rong Jiang

**Affiliations:** Department of Cardiology, Shanghai General Hospital, Shanghai Jiao Tong University School of Medicine, Shanghai, China

**Keywords:** artificial intelligence, lung disease, multisystem abnormalities, neurocognitive ability, pulmonary hypertension

The traditional view of lung disease as an organ-specific disorder has become difficult to sustain. Over the past two decades, evidence has accumulated showing that the lung interacts continuously with the heart, brain, liver, kidneys, and immune system. Chronic hypoxia, systemic inflammation, and metabolic changes in respiratory disease propagate well beyond the pulmonary parenchyma, affecting patient outcomes in ways that isolated organ-based management cannot fully address. Despite this recognition, integrated therapeutic strategies remain underdeveloped, and the mechanisms underlying these multisystem interactions are still incompletely understood.

This Research Topic, “Complex Interplay Between Lung Diseases and Multisystem Disorders,” brings together twenty-six contributions from investigators in Asia, Europe, and North America. The studies span systematic reviews, original research, case reports, and trial protocols, and they examine the lung–system interface from several angles. What follows is a brief overview of the major themes, with emphasis on the studies that advance the field methodologically or conceptually.

## Cardiopulmonary and vascular disease

Several contributions address the well-established but still underappreciated link between lung and cardiovascular pathology. Li et al. report a meta-analysis showing that patients with idiopathic pulmonary fibrosis face approximately twice the risk of ischemic heart disease and thromboembolism compared with controls. The authors go beyond epidemiology to examine shared pathways involving chronic inflammation and endothelial dysfunction, arguing that IPF and cardiovascular disease should be treated as aspects of a single pathological process rather than as unrelated comorbidities. Yuan et al. focus on mitochondrial dysfunction in pulmonary hypertension, tracing how BMPR2 signaling disruption, oxidative stress, and metabolic reprogramming converge at the level of mitochondrial bioenergetics. This mechanistic focus is complemented by Zhao et al. who use eleven years of hospitalization data from Shanghai to show that both extreme heat and moderate temperature deviations independently increase pulmonary hypertension admissions—a finding that has direct implications for patient counseling and public health planning. On the diagnostic side, Fan et al. apply machine learning to chronic thromboembolic pulmonary hypertension in high-altitude pulmonary embolism patients, while Wan et al. develop a noninvasive PECS model for combined post-capillary disease. These computational studies (Li et al.; Liu et al.; Feng et al.) illustrate how algorithmic approaches can handle the complexity of multisystem data more effectively than conventional regression models.

## Neurocognitive and metabolic consequences

Deng et al. report cognitive deficits in interstitial lung disease patients, with measurable impairment in attention, calculation, and orientation. The study is notable for linking these clinical findings to biological mechanisms—specifically, enrichment of neurodegeneration-related pathways in lung tissue transcriptomics. Jia et al. extend this to pulmonary embolism, showing that cognitive dysfunction after embolic events is not merely an epiphenomenon but may reflect genuine central nervous system involvement. On the metabolic side, Qian et al. demonstrate that twelve weeks of structured exercise improves peak oxygen uptake and circulatory efficiency in COPD patients with non-alcoholic fatty liver disease, even when spirometric parameters change little. This suggests that exercise intolerance in COPD is driven more by systemic cardiovascular and metabolic constraints than by pulmonary mechanics alone, and that the presence of NAFLD should not be a reason to withhold rehabilitation.

## Biomarkers, multi-omics, and artificial intelligence

Kuang et al. compare several machine learning algorithms for predicting COPD exacerbations and identify XGBoost as the most robust approach, with a test-set AUC of 0.824. The value of this work lies in its demonstration that machine learning can capture non-linear interactions between inflammatory markers, organ function indicators, and comorbidities without requiring the investigator to specify interaction terms in advance. Burke et al. use integrated miRNA–mRNA network analysis to position miR-182-5p as a potential regulator in COPD, while Zhao et al. identify circTSN as a systemic biomarker responsive to exercise training. Ye et al. explore plasma exosomal markers for lung cancer brain metastasis, and Pös et al. review the emerging field of breath biopsy—cell-free nucleic acids in exhaled breath condensate. Together, these studies make a practical point: static pulmonary function tests are insufficient for assessing multisystem disease, and clinicians will increasingly need to integrate circulating biomarkers, multi-omics data, and algorithmic prediction tools into routine practice.

## Infection, immunity, and rare disorders

Liu et al. develop a nomogram for predicting treatment failure in tuberculosis complicated by diabetes, using readily available clinical and laboratory variables. This has immediate applicability in resource-limited settings where TB–diabetes comorbidity is common and treatment failure carries serious consequences. Dimeas et al. review non-culture diagnostics for pneumococcal parapneumonic effusions, addressing a practical gap as culture yields decline. Kudlay et al. provide a broad review of immunopathological syndromes, and Dai et al. describe a protocol for a randomized trial of the Yiqi Gubiao Pill in tuberculosis-associated obstructive lung disease.

The case reports in this Research Topic are instructive precisely because they describe unusual presentations that challenge standard diagnostic assumptions: disseminated blastomycosis presenting as monoarthritis (Ssentongo et al.), cryptococcal pericarditis in an immunocompetent host (Shu and Zhou), actinomycosis with splenic rupture and hepatic abscesses (Li et al.), and vascular Ehlers-Danlos syndrome presenting as massive hemothorax (Han et al.). These cases serve as reminders that respiratory pathogens and genetic disorders can produce multisystem complications that are easily missed if the clinician maintains a narrow pulmonary focus.

## Procedural and acute complications

Yang et al. report a rare but serious complication of flexible bronchoscopy—severe pneumoperitoneum and thoracoabdominal emphysema—while Fu et al. synthesize evidence on postoperative venous thromboembolism after lung cancer surgery. Both studies highlight risks that bridge procedural technique, coagulation biology, and cardiorespiratory outcomes.

## Concluding observations

Several themes recur across these twenty-six studies. First, the mechanisms linking lung disease to multisystem complications are increasingly understood at the molecular level—inflammation, mitochondrial dysfunction, and fibrotic pathways appear in multiple contexts. Second, diagnostic and prognostic assessment in respiratory disease is becoming more data-intensive, with machine learning and multi-omics offering tools that conventional clinical testing cannot match. Third, therapeutic approaches need to become more integrated; the failure of isolated organ-targeted strategies in conditions like IPF-associated pulmonary hypertension illustrates the limitations of current practice.

We thank the Guest Editors for assembling this collection, the authors for their contributions, and the reviewers for their critical assessments. Volume II of this Research Topic is now open for submissions. We encourage readers to approach these articles not as isolated reports on pulmonary pathology, but as contributions to a broader understanding of respiratory disease as a systemic condition.

